# Future perspectives for treating patients with geographic atrophy

**DOI:** 10.1007/s00417-022-05931-z

**Published:** 2022-12-15

**Authors:** Anat Loewenstein, Omer Trivizki

**Affiliations:** Division of Ophthalmology, Tel Aviv Sourasky Medical Center, Tel Aviv University, 6 Weizmann St, 64239 Tel Aviv, Israel

**Keywords:** Age-related macular degeneration, Complement, Disease progression, Geographic atrophy, Imaging, Vision

## Abstract

**Purpose:**

Geographic atrophy (GA) is a late-stage form of age-related macular degeneration (AMD) characterized by the expansion of atrophic lesions in the outer retina. There are currently no approved pharmacological treatments to prevent or slow the progression of GA. This review describes the progression and assessment of GA, predictive imaging features, and complement-targeting investigational drugs for GA.

**Methods:**

A literature search on GA was conducted.

**Results:**

Expansion of atrophic lesions in patients with GA is associated with a decline in several measures of visual function. GA lesion size has been moderately associated with measures obtained through microperimetry, whereas GA lesion size in the 1-mm diameter area centered on the fovea has been associated with visual acuity. Optical coherence tomography (OCT) can provide 3-dimensional quantitative assessment of atrophy and is useful for identifying early atrophy in GA. Features that have been found to predict the development of GA include certain drusen characteristics and pigmentary abnormalities. Specific OCT features, including hyper-reflective foci and OCT-reflective drusen substructures, have been associated with AMD disease progression. Lesion characteristics, including focality, regularity of shape, location, and perilesional fundus autofluorescence patterns, have been identified as predictors of faster GA lesion growth. Certain investigational complement-targeting drugs have shown efficacy in slowing the progression of GA.

**Conclusion:**

GA is a progressive disease associated with irreversible vision loss. Therefore, the lack of treatment options presents a significant unmet need. OCT and drugs under investigation for GA are promising future tools for disease management.

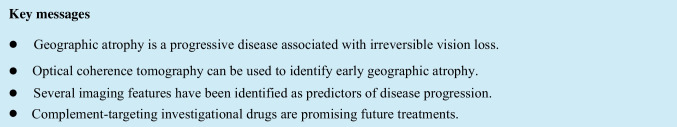

## Introduction

Geographic atrophy (GA) is a late-stage form of age-related macular degeneration (AMD) characterized by progressive degenerative lesions affecting photoreceptors, retinal pigment epithelium (RPE), and choriocapillaris primarily in the macula [1–5]. Although patients with AMD may benefit from supplements consisting of high-dose antioxidant vitamins (C, E, and beta-carotene) and zinc, which have been found to decrease the risk of progression to advanced AMD [6], there are currently no approved pharmacological treatments to prevent or slow the progression of GA [7]. Thus, management largely consists of monitoring disease progression [1]. Nevertheless, early identification of patients with AMD is important because patients may ultimately benefit from drugs that are currently under investigation for GA. This report provides an overview of the progression and assessment of GA, predictive imaging features, and treatments currently on the horizon.

## Disease progression and visual function

### Early and intermediate age-related macular degeneration

Features of AMD observed prior to the development of GA include the presence of medium or large drusen, which are deposits between Bruch membrane and the RPE, as well as AMD pigmentary abnormalities, which have been defined as hyper- or hypopigmentation (within 2-disc diameters of the center of the macula) associated with medium (63 to 125 µm) or large (> 125 µm) drusen and without other reasons for the abnormalities [8]. AMD has been classified into early, intermediate, and late stages [8]. Early AMD is defined by the presence of medium-size drusen without pigmentary abnormalities, whereas intermediate AMD is defined by the presence of large drusen and/or AMD pigmentary abnormalities [8]. Early stages of AMD have been reported to usually be asymptomatic [8]. Individuals with early or intermediate AMD may progress to late AMD, which includes neovascular AMD or GA [8].

### Geographic atrophy

GA is characterized by well-demarcated atrophic lesions in the outer retina that expand over time [1, 4, 9, 10]. Lesions often begin in parafoveal regions and expand to the fovea later in the disease [10–12]. However, there is variability across eyes. In a study of patients with bilateral drusen who were examined annually, the location of incident GA was parafoveal (250–1500 µm) in 61.4%, ≤ 250 µm from the foveal center in 17.5%, and subfoveal in 20.2% [12]. Lesion growth rate varies across studies and individuals and is affected by baseline lesion size (in mm^2^) [13, 14]. In a recent meta-analysis, the pooled mean GA growth rate was shown to be 1.66 mm^2^/year or 0.33 mm/year, and regardless of how GA growth rate was calculated (non-square root transformed, mm^2^; or square root transformed, mm), a significant association was observed with baseline GA area (mm^2^) [13]. Since baseline GA area is 1 of the factors that affect GA growth, square root transformation methods can be used in clinical trials to minimize the impact of baseline GA size [13]. For eyes with GA with foveal sparing, GA progression toward the periphery has been found to be faster than progression toward the fovea [15].

As GA progresses, visual function declines [3, 9, 16, 17]. In a retrospective analysis of the Age-Related Eye Disease Study, the median time to loss of 15 letters was 5.6 years for eyes with non-central GA at baseline (baseline visual acuity: 74.5 letters) and 6.3 years for eyes with central GA at baseline (baseline visual acuity: 52.8 letters) [3]. In addition, some reports have suggested that patients with GA may have deficits in visual-function measures that rely on the fovea even when visual acuity is still relatively preserved (20/50 or better), including low luminance visual acuity, foveal dark-adapted sensitivity, and contrast sensitivity [11]. Furthermore, due to absolute scotomas associated with the GA lesion, patients with GA may have deficits in tasks that require a wide visual field [11, 18].

In the large Chroma and Spectri studies, various measures of visual function were found to worsen over 48 weeks in patients with GA, including best corrected visual acuity, low luminance visual acuity, monocular and binocular maximum reading speed, macular sensitivity, and absolute number of scotomatous points (as measured by mesopic microperimetry), and patient-reported outcomes of visual function (25-item National Eye Institute Visual Function Questionnaire and the Functional Reading Independence Index) [16]; however, correlations between GA lesion area and measures of visual function were found to be weak, especially for best corrected visual acuity [16]. The only visual-function measures that showed moderate correlations (0.4 ≤ Spearman correlation coefficient ≤ 0.59) with GA lesion area at baseline or week 48 were the number of absolute scotomatous points and mean macular sensitivity as measured by microperimetry [16]. Other studies have demonstrated that the relationship between GA lesion area and visual acuity is stronger when the area of analysis is limited to the 1-mm diameter area centered around the fovea than when the total GA lesion area is considered [3, 17]. Although the association between GA lesion size and visual function remains unclear, low-luminance deficit has been shown to be a predictor of future vision loss in patients with GA and good vision (≥ 20/50), which may allow for the identification of patients with a high risk of losing vision [19].

Although previously considered as distinct entities, GA and choroidal neovascularization (CNV) are not mutually exclusive. Eyes with bilateral GA or an eye with GA and fellow eye CNV have a significant risk of developing CNV in the eye with GA [20, 21]. Sunness et al noted that the 2-year and 4-year rate of patients developing CNV in an eye with GA was 6% and 17%, respectively [21]. When broken down by fellow eye, the 2-year rate was 18% and the 4-year rate was 34% when fellow eye had CNV, and 2% and 11% in eyes with bilateral GA [21]. Furthermore, progression to advanced AMD (GA or CNV) in eyes with early or intermediate AMD is increased when there is GA or CNV in the fellow eye [20].

## Assessment of geographic atrophy progression

Several imaging modalities can be used to monitor the progression of GA, in particular color fundus photography (CFP), fundus autofluorescence (FAF), and optical coherence tomography (OCT) [22, 23]. CFP is the historical standard for imaging GA; however, it is limited in the contrast that it provides [22, 24]. GA on CFP appears as sharply demarcated areas of hypopigmentation [22]. FAF provides higher contrast than CFP [22]. Due to the loss of RPE cells and lipofuscin with GA, atrophy on FAF presents as regions of hypoautofluorescence [1, 22]. However, assessment of the foveal region is difficult as the macular pigment blocks blue light, making the signal intensity reduced, especially when the atrophic region is close to the fovea and therefore additional imaging modalities are needed [22]. Spectral domain OCT (SD-OCT) has notable advantages for visualizing GA in that it allows for cross-sectional and en-face images and for 3-dimensional quantitative assessment of atrophy of specific retinal layers [22, 25]. Swept-source OCT has further advantages in that it allows for wider imaging areas and greater detail of the choroid [22].

OCT can be used to identify early atrophy in AMD, as was noted by the Classification of Atrophy Meeting (CAM) group [25]. Identification of early atrophy is important for determining whether investigational therapeutics can reduce GA progression at early stages [25, 26]. The CAM group proposed 4 categories of GA based on OCT criteria, which describe whether the atrophy is incomplete or complete and whether it occurs in the presence of RPE atrophy: (1) complete RPE and outer retinal atrophy (cRORA); (2) incomplete RPE and outer retinal atrophy (iRORA); (3) complete outer retinal atrophy (cORA); and (4) incomplete outer retinal atrophy (iORA) [25]. According to this framework, GA and nascent GA are considered subsets of cRORA and iRORA, respectively, which occur in the absence of choroidal neovascularization [25].

## Imaging predictors of disease progression

Several imaging features have been found to predict the development of GA, including certain drusen characteristics and pigmentary abnormalities. These features are valuable in identifying patients who may benefit from future treatments for GA. In a large study of individuals ≥ 49 years of age, characteristics of drusen that were strongly associated with the development of GA over a 15-year period included soft indistinct drusen, drusen within 500 µm from the foveal center, and a total drusen area > 375 µm in diameter [27]. The presence of reticular drusen, RPE depigmentation, and hyperpigmentation were also strongly associated with the development of GA [27].

Other imaging features, specifically on OCT, have been identified as predictors of disease progression for individuals with intermediate AMD. The presence of intraretinal hyper-reflective foci was found to correlate with progression to either late AMD (cRORA or choroidal neovascularization) or cRORA alone over 1 year [28]. The presence of OCT-reflective drusen substructures, which include low-reflective cores, high-reflective cores, conical debris, and split drusen, was associated with an increase in GA area over 2 years [29]. Furthermore, the presence of heterogeneous internal reflectivity within drusen (ie, calcified drusen) was associated with progression to advanced AMD (neovascular AMD or GA) over 1 year [30]. In addition, the appearance of persistent hyper-transmission defects with a minimum size of 250 µm on en-face swept-source OCT scans was found to increase the risk of progression to GA [31].

Recent studies have also examined imaging features on SD-OCT, specifically, a focus on photoreceptor integrity as photoreceptor loss is indeed 1 of the characteristics of GA [32, 33]. Reiter and colleagues demonstrated a significant association between the junctional zone (area surrounding the GA lesion) and GA growth after 12 months, suggesting that the junctional zone may be a predictor of GA growth [33]. It has also been demonstrated by Pfau et al that qualitatively, progressive photoreceptor degeneration outside GA correlated with GA progression rates [32]. Quantitatively, ellipsoid zone-loss-to-GA boundary distance and thickness of the outer nuclear layer, outer segment, and inner segment were all associated with future progression rates [32]. These studies suggest that by utilizing SD-OCT, photoreceptor loss and thinning could be an outcome measure beyond GA lesion size progression [32, 33].

For individuals with GA, certain features of the lesion have been found to predict faster rates of GA growth. Studies have found that multifocal lesions have higher growth rates than unifocal lesions and that irregularly shaped lesions have higher growth rates than more circular lesions [34, 35]. In addition, extrafoveal lesions have been found to have higher growth rates than foveal lesions [36]. When perilesional FAF patterns have been analyzed, lesions with banded or diffuse FAF patterns have been found to have higher growth rates than lesions with no abnormal FAF pattern or focal FAF patterns [37].

## Complement-targeting treatments for geographic atrophy

Several studies suggest that the complement system, a component of the innate immune system, plays an important role in the development of GA. Various complement genes have been linked to AMD in genome-wide association studies [38, 39]. The odds of GA were estimated to be 2.5 times higher per copy of a common risk allele (*Y402H*) of the *CFH* gene in individuals of European ancestry [40]. In addition to genetic studies, histologic studies have found complement proteins in drusen as well as elevated levels of complement proteins in the outer retinal tissue of postmortem eyes with AMD [41–43]. Furthermore, elevated levels of activated complement products in plasma have been found in individuals with GA [44]. Based on these findings, several drugs targeting the complement system are currently in clinical investigational phases for GA treatment.

Drugs targeting the complement system with phase 3 efficacy and safety data in GA to date include pegcetacoplan and avacincaptad pegol, which are complement C3 and complement C5 inhibitors, respectively [7, 45]. The efficacy and safety of pegcetacoplan administered via intravitreal injections every month or every other month in patients with GA were examined in randomized, double-masked, sham-controlled phase 2 (FILLY; NCT02503332) and phase 3 studies (OAKS and DERBY; NCT03525600 and NCT03525613) [45, 46]. In the phase 3 studies, OAKS found a significant reduction in GA lesion growth (mm^2^) with pegcetacoplan monthly (22% reduction) and pegcetacoplan every other month (16% reduction) compared with sham at month 12, whereas DERBY failed to find a significant reduction [46]. A combined analysis of OAKS and DERBY found a reduction in growth with pegcetacoplan monthly (17% reduction) and pegcetacoplan every other month (14% reduction) at month 12 [46]. Topline safety results showed rates of serious ocular treatment-emergent adverse events of 1.4% for pegcetacoplan monthly, 1.9% for pegcetacoplan every other month, and 0.0% for sham in OAKS; and 0.5% for pegcetacoplan monthly, 0.0% for pegcetacoplan every other month, and 1.0% for sham in DERBY [47]. In the OAKS and DERBY studies, exudative AMD rates determined by the investigators were 5.2%, 4.7%, and 1.4% for pegcetacoplan monthly, every other month, and sham in OAKS, and 6.8%, 3.4%, and 3.4% in DERBY [47, 48]. The efficacy and safety of avacincaptad pegol 2 mg and 4 mg administered through monthly intravitreal injections in patients with GA were examined in a randomized, double-masked, and sham-controlled phase 2/3 study (GATHER1; NCT02686658) [7]. GATHER1 found a significant reduction in GA growth rate (mm) with both avacincaptad pegol 2 mg (27% reduction) and avacincaptad pegol 4 mg (28% reduction) compared with sham at month 12 [7]. There were no reported serious ocular adverse events in avacincaptad pegol groups or sham groups at 12 months [7]. The most common ocular adverse events related to the injection procedure were conjunctival hemorrhage, conjunctival hyperemia, punctate keratitis, and increased intraocular pressure [7]. CNV rates reported by investigators were 9.0%, 9.6%, and 2.7% in the avacincaptad pegol 2 mg and 4 mg and sham groups, respectively [7, 48]. There is an ongoing phase 3, randomized, double-masked, sham-controlled, 24-month, trial that is evaluating monthly and every-other-month intravitreal injections of avacincaptad pegol 2 mg compared with sham in patients with GA (GATHER2; NCT04435366). Recently, the data for the first 12 months, for which patients received monthly avacincaptad pegol 2 mg or sham, became available. GATHER2 met its primary objectives with a significant reduction in observed GA growth rate (mm^2^) with avacincaptad pegol 2 mg (17.7% reduction) compared with sham at month 12 [49]. GATHER2 had a consistent safety profile with GATHER1 with the most frequently reported ocular adverse events were related to the injection procedure. Following a comprehensive surveillance of CNV, the reported incidence of CNV in the study eye at 12 months was 6.7% for avacincaptad pegol 2 mg compared with 4.1% for sham [50]. Post hoc analyses of the FILLY study have demonstrated that pegcetacoplan lowered the rate of iRORA progression to cRORA as well as reduced photoreceptor loss and thinning compared with sham [51, 52]. Similarly, a post hoc analysis of GATHER1 has demonstrated that avacincaptad pegol reduced the progression of drusen to iRORA/cRORA and the progression from iRORA to cRORA compared with sham, and a reduction in the growth of ellipsoid zone degradation [53]. Future analyses of the phase 3 studies for both pegcetacoplan and avacincaptad pegol, as well as other investigational drugs, will provide a further understanding of the complement system in GA and potential treatments for the management of GA.

## Conclusion

GA is a progressive disease associated with irreversible vision loss. Therefore, the lack of treatment options presents a significant unmet need. OCT has been identified as a useful tool for characterizing early atrophy. Furthermore, several imaging features have been identified as predictors of disease progression. Pharmacological agents currently under investigation for GA are being examined for their ability to slow the growth of GA and potentially preserve visual function. Given the irreversible nature of the disease, it is also important to understand whether these drugs can reduce the rates of onset of GA in patients at earlier stages. These investigational drugs are promising future tools for the management of patients with GA.
